# Hyaluronic Acid in Vascular and Immune Homeostasis during Normal Pregnancy and Preeclampsia

**Published:** 2016

**Authors:** M. M. Ziganshina, S. V. Pavlovich, N. V. Bovin, G. T. Sukhikh

**Affiliations:** Federal State Budget Institution “Research Center for Obstetrics, Gynecology and Perinatology” of the Ministry of Healthcare of the Russian Federation, Oparin str. 4, 117997, Russia, Moscow; Shemyakin-Ovchinnikov Institute of Bioorganic Chemistry, Russian Academy of Sciences, Miklukho- Maklaya str. 16/10, 117997, Russia, Moscow

**Keywords:** preeclampsia, hyaluronic acid, glycocalyx, intercellular matrix, glycopathology

## Abstract

Preeclampsia (PE) is a multisystem pathologic state that clinically manifests itself after the 20th week of pregnancy. It is characterized by high maternal and perinatal morbidity and mortality. According to modern concepts, the impairment of trophoblast invasion into maternal spiral arteries, leading to the development of ischemia in placenta, is considered to be the major pathogenetic factor of PE development. Ischemic lesions initiate the development of a systemic inflammatory response (SIR) and endothelial dysfunction, which is the main cause of the multiple organ failure in PE. Some data has appear indicating the importance of a glycans-forming endothelial glycocalyx and extracellular matrix (ECM) for placenta morphogenesis, as well as their role in the regulation of vascular permeability and vascular tone in hypertension disorders and, in particular, PE. Since intact glycocalyx and ECM are considered to be the major factors that maintain the physiological vascular tone and adequate intercellular interactions, their value in PE pathogenesis is underestimated. This review is focused on hyaluronic acid (HA) as the key glycan providing the organization and stabilization of the ECM and glycocalyx, its distribution in tissues in the case of presence or absence of placental pathology, as well as on the regulatory function of hyaluronic acids of various molecular weights in different physiological and pathophysiological processes. The summarized data will provide a better understanding of the PE pathogenesis, with the main focus on glycopathology.

## INTRODUCTION

The main factor that determines the successful course of a pregnancy is the formation of a complete fetoplacental system (FPS) that meets the needs of the developing fetus and regulates the hemodynamic load on the mother’s cardiovascular system. The key moment of FPS formation is the transformation of the uterine spiral arteries into uteroplacental vessels that are formed as a result of trophoblast invasion into the wall of the mother’s spiral arteries. The invasion is accompanied by tissue remodeling, wherein lysis of the elastic muscle components of radial arteries, their replacement by a fibrinoid material, and the formation of broad spiral cavities adapted to an increasing blood flow take place [1, 2]. Adequate FPS formation is achieved thanks to the ability of trophoblast to differentiate into cell populations that exhibit various invasive and locomotor features. The cells of the invasive (extravillous) trophoblast acquire the properties of pseudoneoplastic cells with a high proliferative, invasive and migratory potential, as well as specific expression of surface markers during placentation, which enables FPS formation and promotes the phenomenon of nonrejection [3]. Pathogenesis of PE is associated with impaired cell proliferation and invasion of trophoblast into uterine spiral arteries, morphologically manifested in the development of a small cell invasion and the absence of spiral artery remodeling, which is especially pronounced in early PE (clinical signs appears prior to 34 weeks gestation) [4, 5]. Another factor which is pathogenetically important both for early and late PE (manifestation of clinical symptoms after 34 weeks of gestation) is an excessive systemic inflammatory response (SIR), which results in endothelial activation/dysfunction and immune maladaptation [6]. The clinical manifestations of PE (high blood pressure and proteinuria) are due to these factors.

Cell invasion is accomplished through adhesive interactions between cells and ECM and is regulated by endogenous and exogenous factors: gene expression and biomodulators. Trophoblast cells, on the one hand, share some properties with tumor cells, and, on the other hand, their invasion is strictly determined by the terms of gestation and a tolerable depth of invasion. The ability to invade is determined both by the cell properties themselves (their differentiation, synthesis of proteolytic enzymes and cytokines) and the matrix properties: its structure (forms honeycomb frame for the cells) and regulatory function (contains biologically active molecules and functional groups).

The histology and functional properties of ECM are determined by the severity of SIR; its degree is considered as one of the leading factors that determine, on the one hand, the possibility of tissue remodeling (physiological remodeling in normal pregnancy and pathological remodeling in pathologic pregnancy or oncotransformation) and, on the other, the possibility of intercellular communication (exposed glycans and glycoconjugates change under the impact of inflammatory mediators, which manifests itself in the change in cell and organ functions).

Information on the role of ECM and the molecules that form it in PE pathogenesis is rather limited. The current review describes hyaluronic acid (HA), its function as part of the ECM and endothelial glycocalyx, the distribution in placental structures, and the regulatory effect of HA in the processes of invasion and inflammation.

## FUNCTIONS OF HYALURONIC ACID AS PART OF THE EXTRACELLULAR MATRIX

**Fig. 1:**
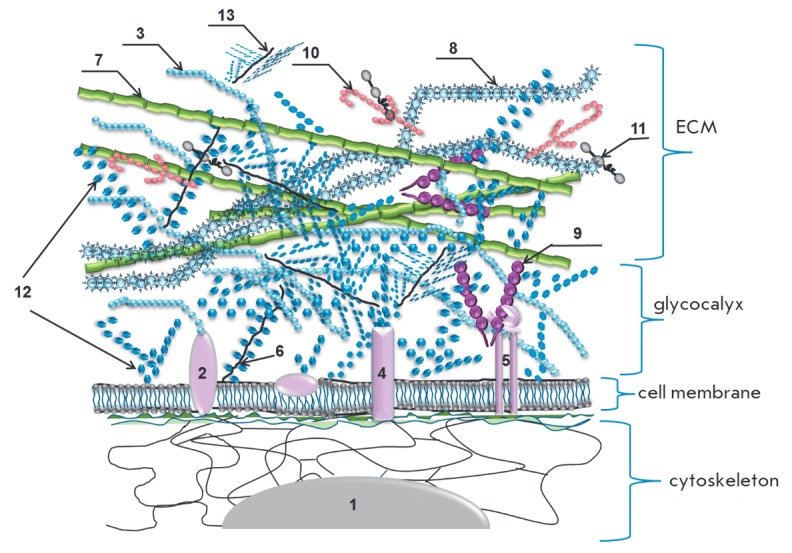
Composition and structure oftheextracellular matrix (ECM*) 1 – nucleus; 2 – hyaladherin; 3 – hyaluronic acid (HA); 4 – glycoprotein; 5 – integrin; 6 – syndecan; 7 – elastane; 8 – collagen; 9 – fibronectin; 10 – laminin; 11 – nidogen; 12 – gel-forming polysaccharides; 13 – small soluble proteoglycan. *ECM – extracellular complex of glycosaminoglycans and (glyco)-proteins both bound to the membrane and integrated into the complex due to carbohydrate-protein and carbohydrate-carbohydrate interactions. ECM has a cellular construction, forms a framework for the cells and the basis for the connective tissue. ECM provides mechanical support for the cells in tissue, intercellular contacts, and cell transport and migration. The border between ECM and glycocalyx for cells in tissue is rather conventinal. The carbohydrate layer adjacent to plasmolemma is considered to be a glycocalyx, whereas the glycosaminoglycan layer located above and including protein molecules is ECM. ECM impairment leads to disorders in tissue organization with changes in organ function.

**Fig. 2:**
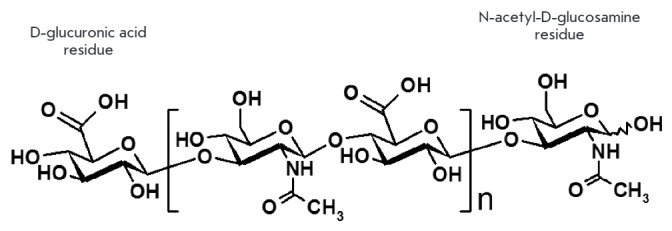
Chemical structure of hyaluronic acid

The extracellular matrix is formed by fibrillar and structural proteins, proteoglycans, and glycosaminoglycans (*Fig. 1*). One of the main components of ECM and endothelial glycocalyx of the cell is HA, which belongs to linear, non-sulfated glycosaminoglycans. The structural unit of HA is a repeating disaccharide consisting of D-glucuronic acid and N-acetyl-D-glucosamine (*Fig. 2*): i.e., HA is a regular polysaccharide. HA is presented *in vivo *mainly in high-molecular (native) form (HMW-HA), while low-molecular-weight HA (LMW-HA) is dominated under SIR (*Table 1*) [7]. HA is found in intracellular compartments and also on the cell surface, in the pericellular and extracellular matrix. Significant amounts of HA are contained in tissues with a high proliferative potential and invasive ability [8]. The stabilization of the dimensional structure of ECM is achieved thanks to non-covalent interactions between HA and small proteoglycans, resulting in the formation of a three-dimensional lattice structure surrounding the cells [9], which acts as a filter and the first line of intercellular interactions: adhesion, migration, and subsequent functional activity. The organizing and stabilizing effect of HA, as part of the lattice structure, plays the key role in the physiology of ECM and glycocalyx [10, 11].

**Table 1:** The biological roles of HA of different molecular weights

HA fractions
**HMW-HA (high molecular weight HA)** molecular mass > 500 kDa, average mass 10^6^–10^7^ Da (~2000–25000 disaccharide units) [9, 73, 74]	**LMW-HA (low molecular weight HA)** molecular mass < 500 kDa (~1000 disaccharide units or less) [74 , 80]	**O-HA (oligomeric HA)** molecular mass 0.75—10 kDa (2 - 20 disaccharide units) [75]
Formation and stabilization of the structure of ECM and glycocalyx; immunologically inert [75, 76]; binds to cell receptors, prevents immune recognition; immunosuppressive action [77]; induction of Foxp3 expression, increases the number of inducible regulatory T-cells [77, 78]; preserves the barrier function of glomerular endothelial barrier [7] HA synthesis suppresses [74]	Functions as a danger signal (danger-associated molecular patterns) [9]; increases permeability of lymphatic capillaries [81], increases vascular permeability [7].	Can regulate various processes both positively and negatively; inhibit endogenous production of hyaluronan [9]
Anti-inflammatory action, inhibition of phagocytosis [77]	Anti-inflammatory action [33]	
Anti-angiogenic effect, prevents transendothelial migration [77]	Pro-angiogenic effect, stimulation of endothelial cell proliferation, adhesion and formation of capillaries [33]	Stimulation/inhibition of angiogenesis, adhesion [9, 80]
Synthesis of immunosuppressive cytokines [77]	Synthesis of pro-inflammatory cytokines [33]	
Stimulates proliferation and inhibits apoptosis of decidual stromal cells in early pregnancy [79]	Stimulates apoptosis of decidual stromal cells in early pregnancy [79]	Stimulation of tumor cell proliferation/ apoptosis [74]
Prevents adhesion and invasion of tumor cells [77]	Stimulation of tumor cell invasion and migration, stimulation of extravillous trophoblast cell invasion [82]	

## FUNCTIONS OF HYALURONIC ACID AS PART OF THE GLYCOCALYX

**Fig. 3:**
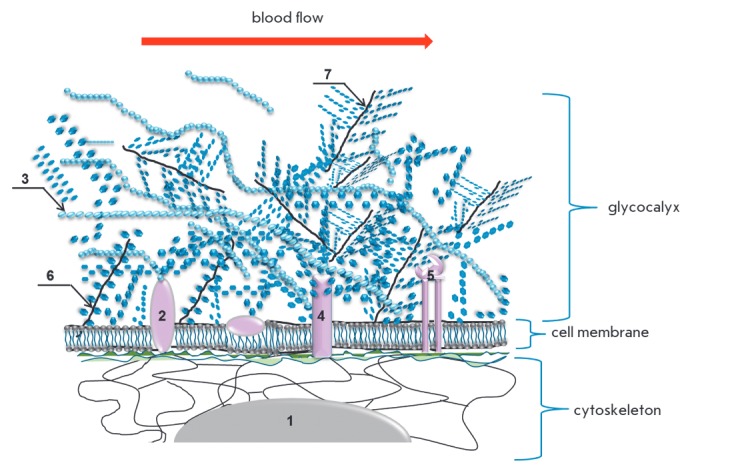
Composition and structure of endothelial glycocalyx* 1 – nucleus; 2 – hyaladherin; 3 – hyaluronic acid (HA); 4 – glycoprotein; 5 – integrin; 6 – syndecan; 7 – small soluble proteoglycan. *Glycocalyx is the external cell envelope associated with the plasma membrane that presents a polysaccharide gel. Glycocalyx executes receptor and protective functions, as well as the function of external signal transducer in circulating cells and cells in contact with biological fluids. Glycocalyx shedding leads to changes of cells receptors.

The endothelial surface layer (ESL) is located on the luminal surface of endothelium and comprises glycocalyx, a complex structure consisting of proteoglycans and glycoproteins that are anchored to the membrane and contain many sialic acid and sulfate residues forming the overall negative charge of the surface of endothelial cells (*Fig. 3*). HA is present in the layer which is in constant dynamic interaction with blood and is formed by secreted and circulating molecules (HA, albumin and α1-acid glycoprotein) [12, 13]. Endothelial glycocalyx is thought to play a key role in the regulation of the physiological and pathophysiological processes taking place in the bloodstream: permeability, tone, coagulation, and the inflammation process [14]. Since the loss of control over the regulation of these processes is significant for PE pathogenesis, one can assume that endothelial glycocalyx can be a central target for the application of the factors that destabilize homeostasis (such as placental ischemia and excessive SIR in PE), thereby leading to clinical manifestations of various severities.

According to current concepts, the regulation of the vascular tone involves cell mechanics and the regulation of mechanical stimuli. Mechanical stimuli are external factors that cause the process of mechanotransduction, i.e., changes in gene expression and cell phenotype due to shear stress (tangential blood flow pressure on endothelial cells), vascular tension, hydrostatic blood pressure, and intercellular contacts [15]. The mechanics of endothelial cells include the properties of individual subcellular compartments (glycocalyx, cell membrane, cytoplasm and nucleus), which are regulated by both mechanical stimuli and biologically active molecules [16, 17]. The structures defining the mechanics of endothelial cells are interconnected: the cell cortex located under the plasma membrane is formed by bundles of microfilaments which are in contact with stress fibrils, microtubules, and intermediate filaments; all the components are organized in a network that fills the cytoplasm and is connected to the nucleus (*Fig. 3*) [18]. Therefore, the function of glycocalyx is to convert the biomechanical and biochemical signals going from the bloodstream into endothelial cells [15], and the effectiveness of its performance is determined by the integrity of the endothelial surface layer.

**Fig. 4:**
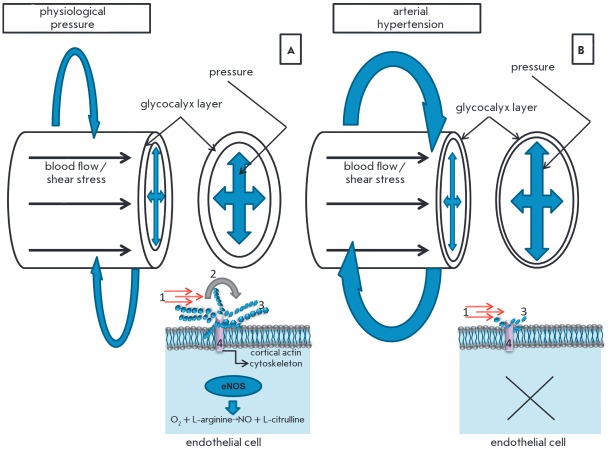
The role of glycocalyx in the regulation of the vascular tone. *A *– Regulation of the vascular tone in physiological conditions. Physiological (intact) glycocalyx is a conductor-transducer of mechanical signals to endotheliocyte: shear stress (1), acting in parallel with the vascular wall (induces internal tension, which is realized as an activation of the signal systems that regulate the vascular tone and permeability); and blood pressure, acting perpendicularly to the vascular wall and imposing a stretching action on all vessel and ECM components. Glycocalyx assumes the mechanical load in the form of local torque (2), disperses it and transduces the signal through proteoglycan chains (3) to the so-called core (anchored in the membrane) proteins (4). The main result is eNOS activation, synthesis of endogenous NO, which causes a vasodilatory effect and the reorganization of the actin cytoskeleton providing the adaptation of intercellular contacts to the mechanical load [15, 21, 134].* B *– Regulation of the vascular tone in arterial hypertension. Shedding or total absence of a glycocalyx layer during pathophysiological processes leads to the mechanical load directly on the apical cell membrane and suppression of endogenous NO production by endothelial cells. As a result, the blood pressure is increased and intercellular contacts are impaired [21, 134].

The physiological effect of shear stress on the intact glycocalyx triggers a response from mechanosensitive cellular components, ion channels, caveolae, integrins, cadherins, growth factor receptors, cytoskeletal structures; and activates the signaling pathways involved in mechanotransduction [19, 20]. The main result of this action is a constant production of endothelial NO synthase (eNO synthase), which regulates the formation of endogenous nitric oxide, a factor that supports the physiological values of the blood pressure in the circulatory system. Under high shear stress values on endothelial cells, which appear with an increasing volume and speed of the blood flow in pregnancy, the functioning glycocalyx provides enhanced eNO synthase activation, thus compensating for the hemodynamic load [21]. Destabilization and desquamation of glycocalyx critically alters the endothelial cell response to mechanical stimuli. Shedding of the glycocalyx layer reduces the mechanosensitivity of endothelial cells, which has a vasoconstrictor effect under increasing blood flow conditions (*Fig. 4*).

A reduction of the glycocalyx layer also manifests itself in a disturbed barrier function of the endothelium, since glycocalyx plays a key role in the regulation of vascular permeability. It has been demonstrated that HA (HMW-HA) binds and inhibits the activity of extracellular serine protease, the enzyme that causes degradation of ECM and glycocalyx [7]. HA fragments of various molecular weights interact with different types of CD44 receptor. HMW-HA negatively regulates vascular permeability by activating the signaling pathways associated with the formation of the cortical layer of actin microfilaments and formation of dense intercellular contacts. LMW-HA positively regulates vascular permeability by inducing the activation of the protease-activated receptor (PAR) of endothelial cells, thus promoting the formation of actin stress fibrils and the disruption of intercellular contacts [22, 23].

An intact glycocalyx is associated with adequate functioning of the glomerular barrier [12]. Enzymatic elimination of HA from the endothelium of glomerular capillaries in mice results in a disturbance of the glomerular filter permeability and the appearance of protein in urine [24].

The integrity of the glycocalyx of tumor cells is assumed to determine their invasive properties, since the shear stress of the interstitial fluid affects cell mechanoreceptors [25, 26]. Experimental modeling of the invasive properties of tumor cells (human kidney carcinoma: cell lines SN12L1 and SN12C with high and low metastatic potential, respectively) in a three-dimensional model of interstitial fluid flow has demonstrated that the impact of hyaluronidase and heparinase on cells blocks the expression of MMP-1 and MMP-2 caused by the pressure of the interstitial fluid, thus reducing the invasive potential of the cells. Glycocalyx degradation, particularly degradation of HA, blocks tumor invasion and negatively regulates the invasive and migratory properties of the cells [27].

Destabilization and shedding of glycocalyx components are promoted by hyperglycemia, endotoxemia, septic shock, oxidized low-density lipoproteins, cytokines, natriuretic peptide, abnormal shear stress, ischemia- reperfusion, as well as by the development of SIR, accompanying, at a certain degree of severity, any pathological process [28, 29]. Shedding/destabilization of the glycocalyx layer due to targeted elimination of HA by hyaluronidases deteriorates the mechanosensitive response of endothelial cells [30].

## BIOSYNTHESIS AND METABOLISM OF HYALURONIC ACID

Under physiological conditions, the processes of biosynthesis and degradation of glycocalyx are balanced [28, 31] and are substantiated by the activity of hyaluronan synthases (HAS1, HAS2, HAS3) and hyaluronidases (Hyal1, Hyal2, PH-20/SPAM1) [9, 32-34]. The genes* Hyal3, Hyal4, *and *Hyalp1 *share a high degree of homology with the genes encoding hyaluronidases Hyal1, Hyal2, and PH-20, but Hyal3 and Hyal4 do not exhibit hyaluronidase activity, while *Hyalp1 *is a pseudogene [35].

**Hyaluronan synthases and disruption of hyaluronic acid synthesis**

HAS1 synthesizes HA of a wide range of molecular weights (500–2000 kDa). HAS2 synthesizes high molecular weight HA (HMW-HA), while HAS3 is responsible for the production of low-molecular HA (LMW-HA) with a molecular mass less than 500 kDa [33, 34]. The enzymatic activity of HAS2 and HAS3 is higher than that of HAS1 [36].

The activity of human genes of hyaluronan synthases is regulated by the genes *HAS1, HAS2 *and* HAS3 *localized on different autosomes. Studies of mice embryogenesis have shown that *HAS1 *is expressed during gastrulation and early neurulation,* HAS2 *is expressed in heart and skeletal structures during the early embryonic period, while *HAS3 *expression is limited to teeth germs and hair follicles [34, 37, 38], suggesting different regulatory elements for transcription control. Disrupted expression of *HAS2* during embryogenesis leads to the embryo’s death;* HAS2-*null embryos were found to exhibit the defects of endocardial cushion, yolk sac and vasculogenesis, as well as disruption of epithelial-mesenchymal transformation [34, 35, 39, 40]. Deletion of *HAS2 *leads to disrupted formation of embryo limbs, including joints [35]. Mice *HAS1-*/*- *demonstrate chronic inflammation of joints with damaged articular cartilage; wherein the HA content in the ECM of knockout mice and wild-type mice is identical. *HAS1 *is believed to be important for HA metabolism in inflammation [41]. Mice with a knockout of the *HAS1 *or *HAS3 *gene are viable and fertile. Increased inflammation is observed in double knockout of these genes in mice on a background of a regeneration of skin wound [36]. However, there are reports on brain size reduction and epilepsy seizures in mice with knockout of the *HAS *gene, with epilepsy being most pronounced in mice with knockout of the *HAS3 *gene [42]. In mammals, all *HAS *genes are expressed in both embryonic and adult tissues, with the expression of *HAS3 *being more pronounced in adult tissues [35]. All *HAS *genes, especially *HAS2,* are overexpressed in carcinogenesis [37].

Increased activity of hyaluronan synthases in Shar- Pei dogs phenotypically manifests itself in skin thickening, skin folds, increased HA skin level, and abnormally high HA blood concentration [43]. The content of HA is also increased in the skin of naked mole rat (*Heterocephalus glaber*), a small burrowing rodent of the African mole-rat family characterized by high life expectancy (about 30 years) and resistance to carcinogenesis. Fibroblasts isolated from the skin of a naked mole rat produce high amounts of HMW-HA. They also demonstrate the presence of an unusual form of HAS2 (Ser to Asn substitution in two conservative parts of the polypeptide chain) and a reduced level of the Hyal2 enzyme responsible for HA degradation [35]. These mammals serve as models for the study of resistance to diseases and aging, in particular (www.naked-mole-rat. org). Noteworthy, a high degree of inbreeding is specific to Shar-Pei dogs and naked mole rats.

In general, the abnormalities of HA biosynthesis have been mostly studied *in vitro *in cells and in animal models. Very limited data suggest a relation between* HAS2 *mutation and the development of a ventricular septal defect in Chinese children [44]. It has also been shown that *HAS2 *is overexpressed in Down’s syndrome [45].

**Hyaluronidases and disruption of hyaluronic acid metabolism**

Expression of *Hyal1, Hyal2, *and *Hyal3 *has been detected in somatic tissues; the expression of *SPAM1 *– in testicular tissue (SP-20 is required for fertilization); and *Hyal4 *– in skeletal muscles and placenta [35, 44, 46-48]. HA degradation can occur both intracellularly in the lysosome and extracellularly. Hyal1 is active in lysosomes, hyaluronidase PH–20 functions on the cell surface as a GPI-anchored protein, and Hyal2 cleaves HA both in lysosomes and the extracellular space [34]. Each hyaluronidase is characterized by a specific localization in different cells and a specific pH range within which they remain active; this leads to the generation of hyaluronic acids of different molecular weights [7].

Rare cases of mucopolysaccharidosis type IX, a genetic disorder of the connective tissue, are associated with the lack of enzymes that degrade HA. Mucopolysaccharidosis type IX biochemically manifests itself in the accumulation of HA in tissues, mainly in the lysosomes of macrophages and rarely in the lysosomes of fibroblasts, as well as by an increase in HA concentration in blood in the absence of the enzyme [35, 49, 50]. Mucopolysaccharidosis type IX clinically manifests itself in craniofacial dysmorphism, growth retardation, swelling, tenderness of the joints and juvenile idiopathic arthritis. The neurological status and intellectual development of patients remains within the normal range [35]. A genetic analysis has revealed homozygosity and mutations in the *Hyal1 *gene, but the lack of pronounced anomalies indicates compensation ofthe Hyal1 function by other hyaluronidases [35].

No generalized accumulation of HA has been found in the tissues of *Hyal1^-^*/*^-^*mice, although they demonstrate pronounced degenerative changes in the knee joint cartilage. Mice *Hyal2^-^*/*^-^*demonstrate skeletal abnormalities, hemolytic anemia, thrombotic microangiopathy, severe cardio-pulmonary failure, and high mortality [51-53]. The consequences of ischemia/reperfusion injury to the kidney in knockout mice proved more severe than in wild-type mice. Knockout mice demonstrated a high level of HA accumulated in the injured kidney, a more pronounced inflammation, and kidney fibrosis [54].

The relation between the expression of HA metabolism genes and the invasive properties of cells, as well as the progression of the disease, remains the most well studied to date. *HAS1 *expression has been revealed at low levels in most normal cells, while *HAS2 *expression is detected predominantly during embryogenesis. The expression of *HAS1 *significantly increases in carcinogenesis, while *HAS2 *and *HAS3 *are overexpressed in aggressive forms of cancer [37]. Cells expressing *HAS2* exhibit the most aggressive properties. The study of the expression of hyaluronan synthases/hyaluronidases in a panel of human cell lines of breast cancer with different invasive properties has showed that highly invasive cells predominantly express isoforms of HAS2 and Hyal2, while less invasive cells produce HAS3 and Hyal3 [55]. Transfection of human breast adenocarcinoma MCF-7 cells, immortalized human HaCat keratinocytes, and a primary culture of mouse epidermal keratinocytes with *HAS3*-containing conjugates demonstrated that increased HA synthesis causes the formation of numerous microvillus-like cell surface protrusions, which form the sites for cell contact, attachment, and migration [56]. In this regard, expression of the erbB2 (HER-2/neu) receptor in the area of microvilli seems important [57]. It is assumed that HA can play a key role in tumor invasion, since there is a direct relation between overexpression of HA and erbB2, which promotes the activation of the erbB2-dependent signaling pathway and indicates the importance of HA for the manifestation of an invasive cell phenotype [56].

## REGULATORY EFFECT OF HA POLYMERS OF VARIOUS SIZES

HA can be found in small quantities in the blood of healthy individuals [28, 31], whereas high levels of HA are found in patients with a chronic kidney disease [58], cardiovascular diseases [59], pulmonary hypertension [60], liver cirrhosis [61, 62], and cancer [63]. There is also evidence of an elevated level of HA in the blood in the PE [64, 65] and HELLP syndromes [66]. The level of antibodies to HA and its structural disaccharide are also elevated in PE [67, 68]. The source of HA in the blood in PE remains unknown: HA is assumed to appear in the blood as a result of maternal endothelial dysfunction [69]; placenta can serve as another source of HA [64, 70].

HMW-HA prevails under physiological conditions, while LMW-HA is mostly found in the inflammatory response and tissue damage [71]. The inflammatory response leads to HA degradation and the formation of fragments of different sizes, which have a multidirectional effect on the function of cells, organs, and systems [29]. The interaction of HMW-HA and LMW-HA with cell membrane receptors induces various signaling pathways that positively/negatively regulate the same processes [72]. A characterization of HA of different weights is presented in *Table 1*.

## RECEPTORS OF HYALURONIC ACID

The functional properties of HA manifest themselves through interaction with its receptors: hyaluronan- binding proteins, or hyaladherins. Specific interactions of HA regulate intercellular adhesion, cell migration, differentiation, HA clearance, signal transduction to cell, and the inflammatory response [83-85]. The most important HA receptors are: RHAMM, the first of the identified receptors, which was discovered both on the cell surface and inside cells (in the cytoplasm and nucleus) and CD44, the main receptor of cell surface HA [37]. HA-RHAMM interaction plays a key role in the activation of signaling cascades through the PDGF receptor, Ser/Thr-kinase, and MAP-kinase Erk [85, 86]. Activation of the intracellular RHAMM receptor causes a reorganization of the cytoskeleton and regulates cell migration and proliferation [37, 87]. HA-CD44 signals also involve the activation of receptor tyrosine kinases (receptors PDGF-β and ErbB2/ Her2), ERM family proteins that provide interaction of the actin cytoskeleton with the cytoplasmic membrane (merlin, ezrin, radixin, and moesin); and the IQGAP1 protein associated with the actin cytoskeleton, the activation of which regulates cell morphology, its motility, adhesion, and cell cycle [34, 37, 88-93]. CD44 is capable of forming a complex with the guanine nucleotide exchange factor Tiam1 [94]. Binding of the complex to HA activates the Rac1-mediated signaling pathway, which also regulates cytoskeleton reorganization [37]. HA metabolism is assumed to be regulated through CD44, since a blocking effect of anti-CD44 antibodies has been shown on endocytosis and HA cleavage *in vitro *[95].

Regulation of HA metabolism is performed by hyaladherins LYVE-1, STABILIN-1, as well as STABILIN-2, the main HA receptor in the liver [34]. Positive regulation of the inflammatory response is observed in the binding of LMW-HA or O-HA with Toll-like receptors (TLR2, TLR4) [33]. Binding with the receptor initiates the MAP-kinase cascade, nuclear translocation of NF-κB, and TNFα production [96]. The function of ECM and glycocalyx structure stabilization is mainly provided by large proteoglycans, ITI-proteoglycans, TSG-6, and SHAP [33, 97]. However, each HA-binding hyaladherin is also involved in the stabilization of supracellular structures. The characteristics of the most studied human hyaladherins are presented in *Table 2*.

**Table 2:** Characterization of human hyaladherins

Receptor	Expression	Function	Source
CD44 – adhesion molecule, main receptor of HA. Isoforms: CD44s (standard), CD44e (epithelial), CD44v (variable)	Leukocytes, erythrocytes, some epithelial cells, brain cells, decidual stromal cells, Kaschenko-Hofbauer cells, placental tissue; overexpression in tumor cells	Mediates the processes of lymphocyte activation, adhesion, migration (lymphocyte rolling and homing), embryonic development, angiogenesis, placental tissue remodeling in placentogenesis, as well as invasion and growth of tumor cells, inflammation. Stabilization of ECM and glycocalyx, biodegradation of HA, induction of CD4+CD25+ T-regulatory cells	[9, 33, 37, 79, 80, 98-102]
LYVE-1 – partial homolog of CD44, main receptor of HA in lymphatic system	Cells of the lymphatic vessels, endothelium of lymphatic capillaries, sinusoidal endothelial cells of liver and spleen, cerebral cortex neurons, macrophages in normal and tumor tissues, placental macrophages, fetal placental endothelium, syncytiotrophoblast	Regulation of cell motility, biodegradation of HA in the lymphatic system. Regulation of HA metabolism in the fetoplacental system. Regulation of dendritic cell adhesion and migration, lymphangiogenesis. Functional activity is regulated by sialylation/desialylation of the O-glycan chains of the receptor	[11, 103-106]
CD168-RHAMM – extra/intracellular HA-binding receptor	Intracellular localization (cytoplasm, cytoskeleton, mitochondria, nucleus); extracellular localization (transported to the cell surface where it is able to bind to HA and activate different signaling cascades); low expression in normal cells	Cell signaling, migration, locomotion and adhesion, angiogenesis. Regulation of mitosis. Localization on the cell surface correlates with tumor progression	[9, 80, 101, 107-109]
ICAM-1 – intercellular adhesion molecule	Inducible expression on endothelium, monocytes, T- and B-lymphocytes, keratinocytes; expressed in inflammation and tumor processes	Regulation of inflammatory process. Mediates leukocyte or tumor cell contact with endothelium for subsequent transendothelial migration or invasion; contact cell interaction in the immune response. Inhibition of type II collagen catabolism	[110-114]
TSG-6 (TNF-stimulated gene 6)	Inducible secreted peptide expressed by many cells upon activation	Anti-inflammatory action: inhibits neutrophil migration in a model of acute inflammation. Serves as a cofactor for other hyaladherins	[33, 29, 72, 115]
TLR2 and TLR4 (Toll-like receptors)	Dendritic cells, monocytes, lymphocytes	Regulation (stimulation/inhibition) of inflammatory process upon receptor binding	[77, 116]
STABILIN-1/2 (scavenger receptors)	Constitutively expressed by macrophages (M2), sinusoidal endothelial cells; expression is increased in inflammation and carcinogenesis	Biodegradation of HA	[11, 117, 118]
CD38 (bifunctional enzyme – combines activities of adenosine diphosphate-ribosyl cyclase (ADP) and cyclic ADP-ribose hydrolase (cADPR)	During ontogenesis on hematopoietic cells at various stages of differentiation; immune cells, vascular smooth muscle and bronchial cells	An enzyme regulating the concentration of intracellular calcium and energetic homeostasis of the cell	[119-122]
HABP1/C1QBP – HA-binding protein-1/ protein binding c1q subcomonent	Peripheral blood cells, macrophages, monocytic dendritic cells	Cell signaling, regulation of complement system activation	[33, 123]
SHAP – serum HA-associated protein	Circulates in blood	Structurally identical to ITIH2, inter-alpha-trypsin inhibitor heavy chain 2. It forms a complex with HA, acts as a cofactor when binding to other receptors (CD44). The level of SHAP-HA complex is an indicator of degenerative changes in the liver and the absence of the matrix of ovulated oocytes	[33]
ITI – proteoglycans: inter-alphatrypsin inhibitors	ECM, glycocalyx	Provides integrity and stabilizes the structure of ECM and glycocalyx	[77, 119, 124, 125]
Large aggregated proteoglycans: aggrecan, brevican, neurocan, versican	Matrix proteoglycans are contained mainly: aggrecan – in hyaline cartilages; neurocan and brevican – in brain tissues; versican – in various tissues, endometrial stroma, especially important for endothelial glycocalyx	Provides integrity and stabilizes the structure of ECM and glycocalyx, carcinogenesis	[33, 126]

## HYALURONIC ACID AND ITS RECEPTORS IN PLACENTA

Among placental tissues, HA is found in the stromal structures of the uterus and placenta, as well as in the angiogenic regions of mesometrial decidua basalis [99], mesenchymal villi, and immature intermediate villi of the placenta [101]. Its involvement in endometrial decidualization has been also shown in mice [126]. The study of the distribution of HA and its receptor CD44 in human placental tissue in physiological pregnancy showed that, in the first half of a pregnancy, HA is highly expressed only in the stroma of mesenchymal villi, whose cells proliferate and differentiate rapidly, providing the development of a placental villous tree. In another type of villi, HA was detected only in the fetal vessels and connective tissue adjacent to the trophoblast, as well as in limited stromal areas of the villi adjacent to the cells of extravillous cytotrophoblast and cell colonies. It is assumed that the significant amounts of HA found in mesenchymal and immature intermediate villi are needed in the capacity of substrate through which mesenchymal cell migration and sprouting of blood vessels take place. Villous stromas of all types are homogeneously stained for HA in mature placenta [101].

HA receptors are also expressed in placental tissue. For instance, the expression of CD44 is detected on decidual cells, lymphocytes localized in the decidua basalis, and cellular elements of endometrial stroma during a normal pregnancy [99]. Invasive extravillous trophoblast express CD44 in the first half of a pregnancy. Increased expression of CD44 positively influences the invasive properties of trophoblast in Matrigel, with HMW-HA inhibiting CD44-mediated invasion and LMW-HA, on the contrary, increasing it [82]. R. Zhu* et al*. have shown that expression of HA and HAS2 by trophoblast in a normal pregnancy is higher compared to early abortion, suggesting the involvement of HA in placental morphogenesis. However, an analysis of the influence of HA of various molecular weights on trophoblast invasion in Matrigel has shown that HMWHA enhances the proliferation and invasive properties of trophoblast, inhibits apoptosis, and activates the PI3K/AKT and MAPK/ERK1/2 signaling pathways in trophoblast, while LMW-HA does not cause these effects. Blockage of the PI3K/AKT and MAPK/ERK1/2 signals inhibits HA-dependent proliferation and the invasive properties of trophoblast [79]. Similar results have been obtained for decidual stromal cells during early pregnancy: the expression of HA, HAS2, and CD44 was lower in abortion than in a normal pregnancy; HMW-HA positively regulated the proliferation, apoptosis, PI3K/AKT- and MAPK/ERK1/2-mediated signals of decidual stromal cells, which illustrates the role of HA and its receptor in decidualization and placentation early in a pregnancy [127].

In early pregnancy, the CD44 receptor is detected in a limited number of Hofbauer cells of the villous stroma and the endothelial cells of small vessels. Increased expression is observed by the 16th week of gestation: the receptor is detected in the intima of fetal blood vessels and connective tissue adjacent to them; limited staining is noted in the cytotrophoblast islands of the basal plate. By the end of a pregnancy, receptor expression is observed in various types of villi; staining was the most pronounced in stem villi. A change in the regulation of the expression of HA and its receptor in placental tissues at different stages of gestation allowed us to presume an active participation of HA in the early morphogenesis of placenta, as well as the important role of CD44 in tissue remodeling during late pregnancy [128].

The HA receptor LYVE-1 was identified in fetal placental endothelium [104] and syncytiotrophoblast [105]. However, its expression was higher than in the mature placenta by 33–34 weeks of gestation [104]. LYVE-1 is also expressed in the population of placental macrophages with the DC-SIGN+CD163+ phenotype localized in the chorionic villi of mature human placenta [105]. Experimental modeling of peritoneal endometriosis in mice showed that the expression of LYVE-1 by the endothelium of lymphatic vessels is increased only after a pregnancy. This effect was absent in treated non-pregnant animals, indirectly pointing to LYVE-1 involvement in angiogenesis [129]. There are no lymphatic vessels in human endometrium; pregnancy causes a rapid induction of lymphangiogenesis in the decidual membrane of the uterus [130]. It is assumed that LYVE-1 participates in the manifestation of an invasive phenotype of trophoblast in the placenta. However, these assumptions are speculative, since there is evidence of an absence of a receptor on the fetal endothelium and endothelium of lymphatic vessels during decidualization [131, 132].

High levels of HA have been found in the area of fibrin deposits in the villi and intervillous spaces in PE [64, 70]. However, there are reports of an absence of differences in the HA content in placental tissues between a normal pregnancy and PE [133]. It should be noted that the distribution of HA and its receptors in patients with early PE remains poorly studied; this complicates the interpretation of the results, since early development of PE in itself is associated with impaired placental morphogenesis.

## CONCLUSION

HA and its receptors are factors that regulate the processes of morphogenesis, epithelial/mesenchymal transformation, tumor metastasis, and tissue remodeling. HA stabilizes endothelial glycocalyx, ensures its integrity and regeneration upon damage; i.e., it maintains vascular homeostasis and provides the barrier function in endothelium. In accordance with the data presented above, one can assume that HA is important during pregnancy, first of all, for placental morphogenesis, and, secondly, for the proper functioning of the regulation of the cardiovascular system, including uteroplacental circulation. Thirdly, since HA regulates the systemic inflammatory response, hyaluronic acids of different molecular weights can have a multidirectional effect on a pregnancy, and even promote pathology. However, despite the proven value of HA in maintaining physiological homeostasis, the role of HA and its receptors in pregnancy remains poorly understood. This applies, primarily, to PE pathogenesis, since the main clinical manifestations of the disease are related to inadequate placentation, excessive systemic inflammatory response, and endothelial dysfunction. The distribution of HA and its receptors in PE, especially in a severe disease, remains poorly studied. To date, the glycocalyx of glomerular and vascular endothelium remains to be studied in cases of a fatal outcome and in an animal model. In addition, the molecular weight of hyaluronic acids in the blood of patients with PE still has to be characterized, and how they affect the disease has not been shown. The study of HA in this context could lead to new discoveries in the pathogenesis of PE.

## Abbreviations

PE: preeclampsia
ECM: extracellular matrix
HA: hyaluronic acid
SIR: systemic inflammatory response
FPS: fetoplacental system
HMW-HA: high molecular weight hyaluronic acid
LMW-HA: low molecular weight hyaluronic acid
O-HA: oligomeric hyaluronic acid
MMP-1: matrix metalloproteinase-1
MMP-2: matrix metalloproteinase-2
